# Molecular Determinants of Human T-lymphotropic Virus Type 1 Transmission and Spread

**DOI:** 10.3390/v3071131

**Published:** 2011-07-12

**Authors:** Michael D. Lairmore, Rajaneesh Anupam, Nadine Bowden, Robyn Haines, Rashade A. H. Haynes, Lee Ratner, Patrick L. Green

**Affiliations:** 1 Department of Veterinary Biosciences, The Ohio State University, Columbus, OH 43210, USA; E-Mails: anupam.1@osu.edu (R.A.); nyb_dvm@yahoo.com (N.B.); haines.106@osu.edu (R.H.); rashade23@sbcglobal.net (R.A.H.H.); green.466@osu.edu (P.L.G.); 2 Comprehensive Cancer Center, The Arthur G. James Cancer Hospital and Solove Research Institute, The Ohio State University, Columbus, OH 43210, USA; 3 Department of Medicine, Pathology, and Molecular Microbiology, Division of Biology and Biological Sciences, Washington University School of Medicine, Campus Box 8069, 660 S. Euclid Ave., St. Louis, MO 63110, USA; E-Mail: lratner@im.wustl.edu (L.R.)

**Keywords:** HTLV-1, human T-lymphotropic virus type-1, transmission, replication, determinants, animal models

## Abstract

Human T-lymphotrophic virus type-1 (HTLV-1) infects approximately 15 to 20 million people worldwide, with endemic areas in Japan, the Caribbean, and Africa. The virus is spread through contact with bodily fluids containing infected cells, most often from mother to child through breast milk or via blood transfusion. After prolonged latency periods, approximately 3 to 5% of HTLV-1 infected individuals will develop either adult T-cell leukemia/lymphoma (ATL), or other lymphocyte-mediated disorders such as HTLV-1-associated myelopathy/tropical spastic paraparesis (HAM/TSP). The genome of this complex retrovirus contains typical gag, pol, and env genes, but also unique nonstructural proteins encoded from the pX region. These nonstructural genes encode the Tax and Rex regulatory proteins, as well as novel proteins essential for viral spread *in vivo* such as, p30, p12, p13 and the antisense encoded HBZ. While progress has been made in the understanding of viral determinants of cell transformation and host immune responses, host and viral determinants of HTLV-1 transmission and spread during the early phases of infection are unclear. Improvements in the molecular tools to test these viral determinants in cellular and animal models have provided new insights into the early events of HTLV-1 infection. This review will focus on studies that test HTLV-1 determinants in context to full length infectious clones of the virus providing insights into the mechanisms of transmission and spread of HTLV-1.

## 1. Introduction

Human T-lymphotrophic virus type-1 (HTLV-1) is a member of the deltaretroviridae, a family of retroviruses which includes both simian T-lymphotrophic virus (STLV-1) and bovine leukemia virus (BLV). Based on epidemiology studies it has been estimated that approximately 15 to 20 million HTLV-1 carriers exist throughout the world, with endemic areas in Japan, the Caribbean, and Africa [1]. In these endemic areas there is a wide range of seroprevalance rates ranging from 0.1 to 30%. After prolonged latency periods (as long as 20 to 60 years), approximately 5% of HTLV-1 infected individuals will develop either adult T-cell leukemia/lymphoma (ATL), or other lymphocyte-mediated disorders such as HTLV-1-associated myelopathy/tropical spastic paraparesis (HAM/TSP).

HTLV-1 is spread through contact with bodily fluids containing infected cells. Contaminated whole blood or whole blood products represent the most common form of HTLV-1 transmission in the United States of America, typically from sharing of needles among intravenous drug users [2,3]. However the more natural route of HTLV-1 transmission is through infected mothers who breast feed their children resulting in the transfer of infected maternal lymphocytes to their infant [4]. Perinatal contamination of the fetus from infected maternal blood occurs, but does not represent a significant mode of HTLV-1 transmission [5]. The transmission of HTLV-1 through sex is a less efficient route of transmission, however male to female transmission via semen is four-times as likely to lead to transmission as female to male [6]. Transfusion of infected blood products remains a major public health concern, and is a principal reason for current blood donor screening procedures particularly in the United States, Japan, as well as other countries [7]. Japan has educated HTLV-1 infected mothers about the possible risks of transmitting HTLV-1 through breast feeding, effectively reducing transmission in endemic regions [8,9].

Mechanisms of how HTLV-1 is transmitted between cells is an active area of research. HTLV-1 is poorly infectious as cell-free virus particles for most cell types. The exception appears to be dendritic cells which can be infected by cell-free HTLV-1 [10]. Well organized cell-to-cell contacts between HTLV-1-infected cells and uninfected T-cells have been described as “virologic synapses” [11,12]. These unique contact points have similar features as immunologic synapses during antigen presentation and appear to be virus-mediated, in part, through Tax [13]. As discussed in subsequent sections of this review, HTLV-1 p12/p8 expression increases T-cell contact through specific adhesion molecules and promotion of cellular conduits appears to enhance cell-to-cell viral transmission [14]. Recently more complex cell surface structures have been described that may protect HTLV-1 particles during cell-to-cell transfer. Glycoprotein-rich aggregates on the surface of HTLV-1-infected cells analogous to “biofilms” suggest unique virus-mediated events may promote successful transmission between cell contacts [15]. Disruption of these structures inhibits cell-to-cell transmission by the virus. The role of specific viral determinants that mediate the formation of these glycoprotein matrix structures remains to be determined.

## 2. HTLV-1 Associated Diseases

Adult T-cell leukemia/lymphoma, in its acute form, is an aggressive T-cell malignancy that typically occurs 20 to 30 years after infection with HTLV-1 [16]. Neoplastic disease associated with HTLV-1 is exhibited in a variety of clinical forms, but is characterized by a monoclonal population of T-cells that express CD3+/CD4+/CD8−/CD25+/HLA-DR+ cell surface markers [17–19]. Approximately 1 to 5% of HTLV-1 infected patients eventually develop some form of ATL after a prolonged clinical latency period (Table 1).

Clinically, ATL occurs in at least four different forms: (1) smoldering (2) chronic (3) lymphoma and (4) acute [17]. Patients afflicted with the acute form of ATL make up approximately 55 to 75% of all ATL cases and present with fever, malaise, skin lesions, lymphadenopathy, leukocytosis and hepatosplenomegaly [17]. To date, there a limited number of successful therapeutic protocols for ATL using standard chemotherapy treatments (reviewed in [20]).

The prolonged and complex interactions between the host and the virus that lead to development of ATL have not been elucidated. HTLV-1 infected neoplastic monoclonal T-cells originate from polyclonal populations of infected T-cells [21]. Selective pressures such as the anti-HTLV-1 adaptive immune response of an infected individual promote an oligoclonal population of infected T-cells with survival advantages to emerge [22]. From this oligoclonal population, a neoplastic T-cell clone emerges typically with a variety of somatic genetic mutations [23,24]. HTLV-1’s transacting transcriptional activator, Tax, plays a major role in the immortalization of these infected T-cells by altering distinct signaling or genetic events such as cell cycle control and DNA repair genes (reviewed in [25]). Tax and HBZ (below) appears to be required for initial immortalization of T-cells promoting the subsequent development of ATL and other lymphocyte-mediated disorders. In addition, evidence is accumulating that implicate the antisense encoded protein, HBZ or its RNA, in T-cell proliferation and perhaps maintenance of transformation [25,26].

In 1985, Gessain *et al.* [27] reported that a group of HTLV-1-seropositive patients in French Martinique suffered from a neurodegenerative disorder called tropical spastic paraparesis (TSP) (Table 1). Osame *et al.* [28] subsequently described a similar clinical disorder in Japanese patients and termed it HTLV-1 associated myelopathy (HAM). The onset of HAM/TSP typically occurs in younger subjects infected with HTLV-1 and is more closely linked to the transfusion of HTLV-1 infected blood products, whereas ATL has been linked to transmission through breast milk of infected mothers [29]. A progressive chronic myelopathy, HAM/TSP mainly affects the thoracic spinal cord and patients often present with urinary incontinence, ataxia, intention tremors and limb paraparesis [30]. The infiltration of HTLV-1 specific CD4+ and CD8+ T lymphocytes into the spinal cord leads to severe inflammation from production of proinflammatory cytokines such as IL-1, IL-6, IFN-γ, and TNF-γ [17]. Accumulation of proinflammatory cytokines leads to demyelination and lymphocytic meningomyelitis. High HTLV-1 antibody titers can be detected in the CSF [31]. The detailed mechanism of HAM/TSP development like ATL has yet to be elucidated. However HTLV-1 proteins utilizing molecular mimicry or acting as autoantigens have been postulated as factors that contribute to the development of HAM/TSP [17,18]. Risk factors for the development of HAM/TSP such as high proviral loads have been linked with the development of HAM/TSP (reviewed in [30]).

A number of other immune-mediated chronic inflammatory conditions are associated with HTLV-1 infection (Table 1) [20,32,33]. However, it is less clear what specific role HTLV-1 infection plays in the initiation or development of these diseases. HTLV-1-associated arthropathy, uveitis, infective dermatitis, polymyositis, chronic respiratory disease, Sjogren’s syndrome, lymphadenitis, and certain acute myeloid leukemias have been associated with HTLV-1 infection (reviewed in [20]). It has been hypothesized that the dysregulation of the immune system in chronic HTLV-1 infection promotes diseases (reviewed in [34]).

## 3. Replication and Organization of the HTLV-1 Genome

**Figure 1:**
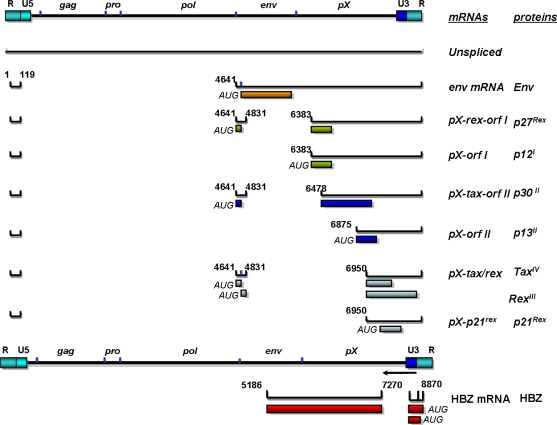
Human T-lymphotrophic virus type-1 (HTLV-1) genome, mRNA, and proteins. The HTLV-1 genome appears on top, the mRNA in the middle, and the protein species on the bottom. The numbers represent nucleotide positions of each exon splice acceptor and donor site.

HTLV-1 is a single-stranded diploid RNA virus that carries genetic information for structural proteins and enzymes (Gag, Env, reverse transcriptase (RT), protease, integrase (IN) (reviewed in [25]). The 3′ end of the viral genome expresses alternatively spliced mRNAs encoding proteins from open reading frames (ORFs) I–IV (Figure 1). The RNA genome is in a ribonucleoprotein complex with the viral protein nucleocapsid (NC). Nucleocapsid along with capsid (CA) and matrix (MA) make up the three proteins produced from the Gag transcript. The env gene encodes for surface unit (SU) and transmembrane unit (TM) proteins. These proteins are responsible for binding and fusion to cellular membranes during viral entry. The enzymatic components of the retrovirus include integrase (IN), reverse transcriptase (RT) and protease (Pro).

The genome of HTLV-1 is approximately 9032 nucleotides long and in its proviral (integrated form) contains two flanking long terminal repeat (LTR) sequences. The LTR’s of HTLV-1 are made up of 3 components, unique region 3′ (U3′), repeated region (R) and unique region 5′ (U5′). These *cis*-acting sequences are critical for viral gene regulation and replication including coordinating transcription initiation and termination, splicing and polyadenylation of mRNA and strand transfer during reverse transcription [35,36]. The U3 contains three imperfect 21 base pair repeats named the Tax response element-1 (TRE-1). The TRE-1 binds multiple transcription factors and is an active site of chromatin remodeling (reviewed in [25,37]).

The pX region contains the regulatory and non-structural genes of HTLV-1. The genes in the pX region are alternatively spliced and made from different initial sites. Open reading frame I and II encode p12 (p8), p30, and p13 [38,39]. Tax, the transacting transcriptional activator and Rex, the transporter of unspliced and single spliced viral RNA, are encoded from ORF-IV and III, respectively [35]. The HBZ gene is encoded from a complementary minus stranded RNA [40]. The expression of viral RNA from primary cells from infected subjects and cells transfected with HTLV-1 molecular clones indicates a two-phased pattern with tax/rex mRNA preceding expression of other transcripts and differential distribution of RNA species between cytoplasmic and nuclear compartments [41].

## 4. Structural Proteins of HTLV-1 and Their Influence on Viral Particle Assembly and Transmission

**Figure 2:**
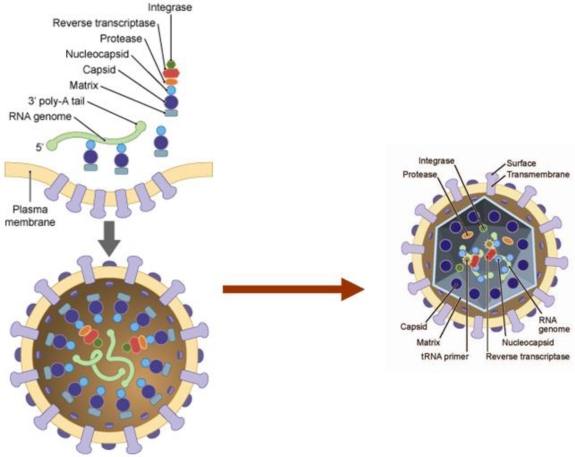
HTLV-1 assembly and incorporation of viral components (left) and fully developed mature virion following budding from cell membrane (right).

HTLV-1 Gag (group specific antigen) or p55 is produced as a single precursor polyprotein. The polyprotein is myristylated, post-translationally, and targeted for the inner lipid plasma membrane of the cell. At the inner membrane of the plasma membrane Gag is cleaved by viral proteases into its functional units: CA (p24), NC (p15) and MA (p19). Capsid interacts with itself to form the inner core of the virion. Nucleocapsid interacts with the genomic RNA inside the inner core of the virion. The proper spatial and temporal events of viral assembly and budding play a critical role in the ability of HTLV-1 to be transmitted from one cell to another (Figure 2).

In contrast to HIV-1 Gag, the interaction of HTLV-1 MA appears to be independent of plasma membrane phospholipid, phosphatidylinositol-(4,5)-bisphosphate [PI(4,5)P(2)] used by HIV-1 in particle assembly [42]. HTLV-1 MA contains a PPPY domain that assists in virus budding by targeting cellular proteins Nedd4.1 and Tsg101 [43–45]. In addition to assisting in virus budding and assembly, MA appears to have a role in cell-to-cell transmission of the virus [46,47]. Utilizing an infectious molecular clone of HTLV-1 (ACH) [48], serine 105 of MA has been shown to be a target of the kinase ERK-2 influencing budding efficiency and viral particle release [47]. Thus, like other retroviruses, the phosphorylation of HTLV-1 L-domain proteins appears to be important in regulation of viral budding and thus cell-to-cell transmission.

Using biochemical approaches and *in vitro* assays, HTLV-1 NC has been documented to function poorly as a nucleic acid chaperone and thus differs from other retroviruses such as HIV-1 [49,50]. In addition, HTLV-1 uses a C-terminal peptide region of NC to block the action of the host restriction factor ABOBEC3G [51]. Future studies using infectious molecular clones of HTLV-1 are needed to test the ability of specific mutations in the key NC motifs that mediate RNA binding and interactions with host restriction factors to understand how they influence the transmission and spread of HTLV-1 *in vivo*.

Protease is produced from ribosomal frame shifting initially as an immature form that is inactive until self cleavage activates the protease after viral budding [52,53]. Reverse transcriptase and IN are generated from proteolytic cleavage of the Gag/Pol precursor polyprotein. Reverse transcriptase is responsible for transcribing the RNA template and IN acts as a catalyst in the integration of the dsDNA viral template into the cellular genomic DNA [54].

The HTLV-1 envelope protein (Env) is maintained among isolates and env variability ranges from 1 to 8% [55–57]. HTLV-1 Env is a 488 amino acid protein synthesized as a polyprotein precursor (gp62), which is subsequently glycosylated and cleaved into two proteins, surface unit gp46 (SU) and transmembrane gp21 (TM) [58,59]. SU is required for entry into the target cell by mediating specific attachment to cellular receptors (below), while the TM supports fusion between viral and cellular membranes to allow viral entry.

HTLV-1 SU is a 312 amino acid protein. The C-terminal half of SU is highly antigenic and is recognized by serum antibodies from approximately 95% of HTLV-1 infected individuals [57]. A major target of neutralizing antibodies is focused on amino acids 187 to 196 of SU [57,60–62]. Early studies using site directed mutagenesis demonstrated functional domains within SU involved in intracellular maturation, syncytium formation, and the association between SU and TM [57,60,63,64]. Subsequent development of a cell transmission assay allowed for separation of fusion events from infectivity events [65,66].

Through a variety of techniques, specific protein motifs of HTLV-1 Env have been defined in terms of their ability to interact with cellular proteins important in cell fusion events. The HTLV-1 TM contains YSLI amino acid sequences that represent consensus YXXP motifs, known to interact with cellular adaptor protein complexes, and a PDZ-binding motif (ESSL) at the C terminus of Env. Alterations of the YSLI motif increased Env expression on the cell surface and increased cell fusion activity, whereas mutations of the ESSL motif reduce Env expression in cells [67]. The human homologue of the *Drosophila* Dlg tumor suppressor (hDlg), a scaffold protein important at cell adhesion sites, is a binding protein with HTLV-1 Env through a PDZ domain and is co-expressed in specific regions of T-cell contacts [68]. RNA interference-mediated knockdown of Dlg1 reduces HTLV-1-mediated syncytium formation apparently by interfering with Dlg1 induced clustering of GLUT1, a cellular receptor for HTLV-1 [69].

Transient transfections of HTLV-1 *env* plasmids with specific mutations in the ACH molecular clone have verified key Env determinants in context to replicating virus [70]. Specific point mutations in env in ACH (ACH.75, ACH.95, and ACH.195) were compared for their ability to elicit antibody responses and proviral loads in a rabbit model of infection [71]. These mutations were within regions predicted to be important for binding of SU to the viral receptor based on syncytium assays or, in the case of ACH.195, in a major target for neutralizing antibody responses [60,62]. These mutations while replication competent, elicited decreased or altered antibody responses in infected rabbits [71]. Mutations that affected Env at position 75 resulted in rabbits developing higher proviral loads than wild type ACH.1 and ACH.95 groups. These data support previous reports of the importance of these regions in SU (amino acids 187–196) in immunogenicity and viral spread *in vivo*.

HTLV-1 SU and TM form as heterodimers at the surface of virions and are responsible for initiating binding, fusion with target cell and entry. The mechanism of action that facilitates cell-to-cell transmission of the HTLV-1 is not resolved, but recently several groups have reported data on three main cellular receptors: glucose transporter (GLUT-1), heparin sulfate proteoglycans and neuropilin-1 [72–82]. Previous studies have shown GLUT-1 to be involved in envelope mediated cell-to-cell fusion [77]. Heparin sulfate proteoglycan binds virus particles on cell surfaces and facilitates entry [83]. In addition to being the main receptor, removal of heparin sulfate proteoglycan from primary lymphocytes significantly reduced binding of SU. Neuropilin-1 is part of the immunological synapse and is a binding partner of Env [80]. Ectopic expression of neuropilin-1 significantly increased HTLV-1 Env-dependent syncytium formation [80]. Further studies will be required to identify specific envelope motifs that both alter receptor binding and influence viral transmission and spread *in vivo*.

## 5. Regulatory Proteins of HTLV-1

### 5.1. Tax

HTLV-1 Tax (Transcriptional Activator of pX region) is a 353 amino acid, 40 kDa phosphoprotein translated from a doubly-spliced mRNA from the ORF IV (reviewed in [25,37,84]. Tax is predominantly a nuclear protein, however it can translocate to the cytoplasm through use of a nuclear export protein [36,85,86]. Tax is responsible for initiating viral transactivation from the LTR of the provirus by binding the GC-rich regions of the TRE-1, within the U3 region of the LTR [87–90]. From the TRE-1, Tax can stabilize the CREB/ATF (activator of transcriptional factors) dimers, which are part of the transcriptional machinery needed for viral gene expression [91,92]. Tax also can recruit and bind CBP/p300 to the TRE-1. Phosphorylation of CREB by PKA leads to recruitment of CBP/p300 in normal cells; however in HTLV-1 infected T-cells Tax can bypass PKA-mediated phosphorylation of CREB. The ability of Tax to recruit and stabilize CREB-CBP/p300 and other factors like P/CAF (CBP/p300 associated factor) allows for efficient transcription of the provirus [93,94].

Tax can also bind TRE-2 in the LTR, which is located central and proximal to TRE-1. Tax recruits transcriptional co-activators like the aforementioned P/CAF and p300 (via KIX domain), Ets family transcription factors (Ets-1, -2, Elf-1, Tif-1) and c-Myb transcription factors to the TRE-2 region [95]. Tax can also bind the basic region of cellular basic leucine zipper transcription factors (bZIP), which aid in DNA binding. The presence of TRE-1 and -2 allows for Tax to mediate a number of processes and facilitate viral transcription bypassing cellular signals.

Tax can activate expression of cellular genes including: (1) CREB/ATF, (2) NF-κB, (3) AP-1 and (4) SRF that influence cellular signaling pathways. These signaling pathways are responsible for the expression of multiple cytokines including: IL-1, -2, -2Rα, -3, -4, -6, -8, GM-CSF, and TNF α and β [96]. Transcription factors like c-Myc, c-Fos, c-Sis, Erg-1, c-Rel, and Lck are also influenced by expression of Tax [97]. Apoptosis and DNA repair genes like Bcl-X_L_, Bax and PCNA (proliferating cell nuclear antigen) respectively are also affected by Tax expression [98–100]. The transforming ability of Tax is most likely attributable to its influence over the expression of these important cellular genes. The development of ATL serves as a model of how an oncogenic viral protein can indirectly lead to immortalization (IL-2 dependent proliferation) and transformation (IL-2 independent clonal expansion) of T-cells.

The molecular mechanisms that lead to development of ATL have not been completely elucidated to date, however it is clear that Tax plays a pivotal role. Tax facilitates the translocation of NFκB into the nucleus and is responsible for activating transcription of genes that favor cellular proliferation and T-cell survival. Tax can bind p50, p52, p65, and c-Rel NFκB family members [101,102]. Most notably, Tax can bind IκBα, an inhibitor of NFκB nuclear translocation. The association of Tax and IκBα destabilizes the IκBα/β/γ-NFκB complex and allows for NFκB translocation [103]. The IκK complex is phosphorylated and subsequently ubiquinated and degraded by the proteosome. NFκB can then activate prosurvival and anti-apoptotic genes that promote cell survival and replication despite cellular signals that might favor apoptosis. The promotion of cellular replication in spite of accumulation of genetic defects and apoptotic signals in the cell contributes to the transformation of lymphocytes and the development of ATL.

While the role of Tax in cell gene expression, proliferation, and transformation have been extensively studied, the role of Tax in viral transmission is less clear. Presumably, HTLV-1 would not be able to accomplish viral replication and spread without Tax function, but specific Tax determinants in viral transmission and spread are problematic to study. Infectious clones of the virus that have Tax mutations would fail to enhance needed viral gene expression during early stages of cell-to-cell transmission. Interestingly, prostaglandins enhance viral expression via the HTLV-1 LTR through the protein kinase A signaling and Tax transactivates a promoter for cyclooxygenase 2, a prostaglandin synthetase, and induces PGE(2) expression in peripheral and cord blood mononuclear cells [104]. This reciprocal interaction has been postulated to promote viral transmission *in vivo*.

Tax involvement in promoting cell adhesion and thereby cell-to-cell transmission has been reported. Significant correlation exists in cell lines comparing expression of HTLV-1 Tax and CCL22, a CCR4 ligand in HTLV-1-infected T-cells, suggesting an active role of Tax in selective CD4+ T-cell viral transmission [105]. This conclusion is supported by transient Tax expression in an HTLV-1-negative T-cell line that induced CCL22 promoting CCR4 redistributed to cell contact points during *in vitro* transmission, and chemotaxis assays. Thus, HTLV-1-infected T-cells may selectively attract CCR4+CD4+ T-cells [105]. Similarly, Tax induced enhancement of ICAM-1 on the surface of T-cells has been shown to facilitate the formation of viral synapses and therefore may contribute to T-cell tropism and viral transmission [106]. Interestingly the unique cellular microenvironment during HTLV-1 milk-borne transmission may favor virus expression. Lactoferrin, a major milk protein, appears to enhance HTLV-1 replication by enhancing HTLV-1 LTR promoter activity (presumably in context to Tax transactivation). Conversely, the viral infection may enhance the expression of lactoferrin in the mammary gland environment [107].

The detailed mechanisms of how specific Tax induced alterations of the host influence viral transmission and spread wait specific testing of tax mutations in context to full length and infectious viral clones.

### 5.2. Rex

Rex is a 27 kDa phosphoprotein encoded by ORF III of the pX region [108,109]. Rex contains multiple functional domains including a RNA binding domain, nuclear localization sequence (NLS), nuclear export sequence (NES) and a multimerization domain [110].

Unlike Tax, Rex regulates viral gene expression only post-transcriptionally and is responsible for regulating expression of viral RNA. Rex facilitates nuclear transport of unspliced and singly spliced mRNAs (Gag, Env, Pol) into the cytoplasm [111]. The presence of the Rex response element (RxRE) in the U3/R 3′ LTR allows for Rex to bind all viral encoded RNAs [112]. Rex, like HIV-1 Rev, appears to utilize the CRM1/exportin pathway [113,114]. In addition, a 21 kDa Rex-like protein is produced in HTLV-1 infected cells, but lacks a nuclear localizing sequence (NLS) in the N terminus and when expressed in cells inhibit RNA shuttling [115]. The role of the 21 kDa form in the natural infection remains unclear.

The essential role of Rex for viral infectivity has been confirmed using molecular clones with selectively mutations in the rabbit model [116]. A Rex-deficient HTLV-1 full length viral clone (HTLVRex-) was used to provide the first direct evidence that functional Rex expression is not required for *in vitro* immortalization by HTLV-1, but was critical for optimal viral transmission *in vivo* [116]. These results suggest that defects in the ability of Rex to promote unspliced and single spliced RNA to traffic are important for optimal viral spread. Construction of reciprocal recombinant infectious molecular clones of HTLV-1 (ACH) and HTLV-2 (pH6neo) indicated that tax and rex genes do not contribute to transformation tropisms preferences [117]. A more refined study of Rex using point or specific motif deletions in context to infectious clones will be required to understand the detailed mechanism of how Rex influences viral transmission.

## 6. Nonstructural Proteins of HTLV-1: Essential Role in Viral Spread and Transmission

The pX region of the HTLV-1 genome also contain ORF I and II, which encode four nonstructural proteins (Figure 1). Alternative splicing of ORF I and II yields p27, p12, p30 and p13 [38,118,119] All of these spliced mRNA’s share a common 1st exon nucleotide (nt.) 1–119 in the R region of the 5′ LTR. Doubly spliced mRNAs encode 2nd exons that start at either nucleotide 4641 or 4658 and end at nucleotide 4831. There are numerous splice acceptor sites for the alternatively spliced mRNAs of ORFs I and II. The 3rd exons for doubly spliced mRNAs such as p27 and p30 have splice acceptor sites at nt. 6383 and 6478, respectively. The second exons for singly spliced mRNAs such as p12 and p13 have splice acceptor sites at nt. 6383 and 6875, respectively.

Initial studies examining the importance of the nonstructural genes suggested they were dispensable *in vitro* [120], however recent studies have supported the role of these nonstructural genes in the transmission and spread of the virus *in vivo* [14,116,121–126]. The nonstructural genes encoded from ORFs I and II are vital for viral infectivity, maintenance of the virus life cycle and proviral loads *in vivo*, as well as host cell activation and regulation of viral gene transcription [121–123,127–132]. The genes from ORF I and II can be detected in HTLV-1 positive cell lines and in patients (asymptomatic, ATL and HAM/TSP patients) [118,133]. Even though detecting the actual proteins has been difficult, mRNA has been detected by RT-PCR and QC-PCR [119]. Patients also possess antibodies and cytotoxic T-cells (CTLs) directed against these proteins [134,135]. ORF I genes appear to be expressed 100–1000 times less than ORF III and IV genes and ORF II genes are expressed 500–2500 times less than ORF III and IV genes [136]. The huge discrepancy in expression might explain why these proteins are difficult to detect and suggest that they might be regulated differently than Tax and Rex.

### 6.1. p12 and p8

HTLV-1 p12 is a 99 amino acid hydrophobic rich protein, rich in leucine (32%) and proline (17%) [119]. p12 has several predicted amino acid regions or motifs which are responsible for a number of its important functions in the cell. p12 contains two putative transmembrane domains (amino acids 12 to 30; 48 to 67) and two leucine zipper motifs [137]. The predicted leucine zipper motifs form α helices in the protein. Together the putative transmembrane domains and the leucine zipper motifs contribute to the ER localization and dimerization of p12 [138,139]. In addition to the aforementioned motifs p12 contains four putative SH3 binding (PXXP) motifs and one conserved PSLP(I/L)T motif [140]. The proline rich Src homology-3 (SH3) motifs are predicted to be responsible for binding cellular signaling proteins. The first (amino acids 8 to 11) and third (amino acids 70 to 74) SH3 binding motifs are highly conserved among different HTLV-1 strains. The conserved PSLP(I/L)T motif is homologous to the calcineurin binding PxIxIT motif present in Nuclear Factor of Activated T-cells (NFAT) [141]. This sequence has the capacity for calcineurin binding in the cell. Calcineurin and NFAT represent a few of the many Ca^2+^ signaling proteins, transcription factors, and ER/*cis*-Golgi associated proteins in the cytoplasm that p12 interacts with to influence gene expression infected T lymphocytes.

In addition to calcineurin and NFAT, calreticulum and calnexin bind p12 and mediate Ca^2+^ regulated pathways in the cell. These ER associated proteins are involved in regulating Ca^2+^ storage and regulating Ca^2+^ signaling, respectively. Calreticulum is a chaperone protein responsible for retaining the MHC class 1 molecule folded in the ER along its maturation pathway. Calnexin assists in proper protein folding of glycoproteins that enter the secretory pathway. The modulation of NFAT activation and thus T-cell signaling is accomplished through interactions between Ras/MAP kinase by p12. When stimulated by phorbol ester and PMA, p12 can activate NFAT [128]. p12 accomplishes this through releasing intracellular Ca^2+^ from ER storage and by increasing Ca^2+^ entry into the cell [142].

HTLV-1 p12 has numerous effects on cell signaling when expressed exogenously. Exogenously expressed p12 can affect T-cell signaling as well. p12 expressed exogenously can bind IL-2R β and γ subunits [143]. IL-2 expression is increased via NFAT activation in a Ca^2+^ dependent manner in Jurkat and primary T lymphocytes. In turn, this leads to a reduced dependency on IL-2 for T-cell activation in the presence of p12 [144]. In addition to Ca^2+^ signaling related proteins, p12 can also bind vacuolar H+-ATPase and immature peptides of MHC class I, which leads to their proteosomal degradation [145,146]. The proteosomal degradation of MHC class I molecules is predicted to lead to a lower percentage of viral peptides being expressed on the surface of infected cells in the context of an MHC class I molecule [147]. With decreased presentation of viral peptides on MHC class I molecules, infected cells may avoid detection from the HTLV-1 specific adaptive immune response. To test the influence of ORF1 expressed proteins in cell transmission, an established T-cell line immortalized with an HTLV-1 molecular clone, which does not express ORF 1 mRNA, was transduced with a lentivirus vector expressing p12 [148]. In this study, p12 expression conferred a selective growth advantage *in vitro* and increased the colony formation of human T-cells in soft-agar assays. IL-2 stimulation and p12 expression significantly increased the rate of syncytium formation, suggesting a novel role for IL-2 signaling and Jak activation in HTLV-1 virus transmission [148].

HTLV-1 p12 has been demonstrated to be proteolytically cleaved to create a smaller protein, p8. This protein apparently serves a different role and acts to increase T-cell contact through LFA-1 clustering thereby enhancing the cellular contacts among T-cells to enhance viral transmission, while anergizing T-cell signaling [14]. The ability of p8 to decrease T-cell activation is likely mediated through inhibiting proximal T-cell receptor signaling at the immunological synapse where it decreases phosphorylation of key signaling proteins in a LAT-dependent mechanism [14,149].

The ability of p12 to induce LFA clustering on infected T lymphocytes was previously demonstrated and hypothesized to increase the efficiency of cell-to-cell spread of the virus [127]. The processing of p12 into p8 may account for the influence of the ORF1 encoded proteins on LFA-1 clustering on the cell surface and the formation of cellular conduits [14]. Equally as plausible is the ability of p12 to induce calcium-mediated LFA-1 clustering on the surface of T-cells, a known mechanism of LFA-1 functional activation [150–152].

The first evidence that pX ORF1 was important for viral transmission was demonstrated in the rabbit model using a splice acceptor site mutant of the ACH infectious molecular clone [121]. Ablation of the acceptor splice site through the deletion of four nucleotides was associated with a reduction of viral infectivity *in vivo* [121] and *in vitro* in non-stimulated T-cells [123]. The deletion of this p12 acceptor splice site would also introduce a frame shift in the HBZ antisense ORF, resulting in the deletion of the last 24 amino acids of HBZ [153]. However the replication capacity of subsequent specific HBZ mutants in context of molecular clones did not result in complete reduction of infectivity as observed in ORF1 splice mutants [154]. Thus, the rabbit model was able to detect selected HBZ mutations and demonstrated that these were different in viral spread compared to ORF 1 mutants. The alteration of ORF I splice sites did not disrupt the expression of Tax, Rex, and structural proteins such as Env in expression in ACH.p12 [128]. A recent study tested a variety of HTLV-1 mutant molecular clones for their ability to replicate in dendritic cells and *in vivo* in rabbits and macaques [126]. In this study, mutations to ablate p12, p30, and HBZ were introduced in the *Cla*I/*Sal*I cassette from the HTLV-1 molecular clone pBST that encompasses the *orf-I* and the *orf*-*II*. The molecular clone pACH was cleaved at the *Cla*I/*Sal*I to generate the backbone for the construction of all viral mutants. Rabbits inoculated intravenously with these mutant clones had reduced viral loads at 16 weeks post inoculation before recovering to “wild type” control level. Dendritic cell cultures from macaques infected with these mutant clones had reduced viral replication parameters suggesting the importance of this cell type in early viral transmission [126]. Small groups of macaques inoculated with these same mutant molecular clones also exhibited limited viral expression [126] confirming the importance of p12/p8 and p30 expression for viral transmission.

### 6.2. p13

HTLV-1 pX ORFII encodes for p13 from a singly spliced mRNA [155]. The viral protein is predominantly localized in the nucleus and mitochondria of transfected cells [133,139,156,157]. Ectopic expression of p13 affects structure and disrupts the inner membrane potential of mitochondria [158,159]. The mitochondrial targeting signal (MTS) of p13, which allows the viral protein to target the mitochondria, is a predicted α-helix that is arginine rich and amphiphatic [157,158]. The incorporation of p13 into the inner mitochondrial membrane causes morphological changes such as swelling and a loss of inner membrane potential (Δψ) [158]. These alterations of the inner mitochondrial membrane change energy production, redox status and induce apoptosis in cells [158]. p13 influence on cell proliferation *in vitro* is dependent on the stage of cell transformation. The viral protein suppresses Ras-dependent tumor explants in mice likely through modulation of cellular metabolism [160]. The ectopic expression of p13 causes Jurkat T-cells to be sensitive to caspase-dependent, ceramide- and FasL-induced apoptosis. A farnesyl transferase inhibitor that prevents post-translational modification of Ras blocks this suppressive effect of p13 [161]. Importantly, an infectious molecular clone of a HTLV-1 with a selective mutation that prevents the translation of p13, without affecting RNA splicing, failed to establish viral infection in a rabbit model [162]. Interestingly, primary T-cells that express p13 are activated, while causing transformed cells to be sensitive to reactive oxygen species [163]. Collectively these studies indicated that p13 has the ability to modulate cell survival via Ras-mediated cell signaling and has an essential role early virus transmission and in virus persistence. p13 interacts with farnesyl pyrophosphate synthase (FPPS), which is involved with synthesis of FPP substrate and is required for prenylation of Ras and subsequent activation of Ras [164]. This interestingly resembles the action of a protein in bovine leukemia virus, G4 (a nonstructural protein) which acts in a similar fashion as p13 in the mitochondria [165].

### 6.3. p30

HTLV-1 p30 is expressed predominantly in the cell nucleus and found primarily in the nucleolus [139]. In the nucleolus, p30 interacts with the large ribosomal subunit L18a which modulates internal initiation of translation [166]. The bipartite NLS of p30 is contained between amino acids 71–98 [156]. In addition to this bipartite NLS, p30 contains a localization/retention domain at amino acids 73–78 and 91–98 [166]. p30 also contains serine and threonine rich domains that share partial homology to the POU transcription family members activation domains such as OCT-I/II, Pit-1, and POU-1 [133].

p30 decreases reporter gene activity of HTLV-1 LTR and CRE driven reporter genes [132]. The transcriptional activity of p30 may be in part determined through its ability to compete with CBP/p300 with Tax via the KIK domain [130]. Competition between Tax and p30 for CBP/p300 complex on the TRE determines if there is Tax mediated viral gene activation or p30 mediated viral gene repression [130]. p30 also alters viral gene expression post-transcriptionally via its ability to bind Tax/Rex mRNA in the nucleus and prevent its exit into the cytoplasm for translation [167,168]. This post-transcriptional effect of p30 is likely mediated via a ternary ribonucleoprotein complex on select viral transcripts [169]. p30 appears to bind specifically to Tax/Rex mRNAs expressed from molecular clones and not cDNA [167,170]. Jurkat T-cells transduced with a lentiviral vector expressing p30 caused a delay at the G2-M phase of the cell cycle [171]. These data suggest that p30 acts in a prosurvival role in the face of genotypic mutations induced by HTLV-1 replication and Tax expression [171]. Ectopically expressed p30 binds to cellular ataxia-telangiectasia mutated (ATM) and REGγ (a nuclear 20S proteasome activator) [172]. In conditions of genotoxic stress p30 expression was associated with reduced levels of ATM and increased cell survival. These data suggest that HTLV-1 p30 interacts with ATM and REGγ to increase viral spread by facilitating cell survival. In addition, interaction of p30 with Myc-Tip60 complex modifies Tip60 mediated transcription and may promote cellular transformation [173]. HTLV-1 p30 has been recently demonstrated to inhibit conservative homologous DNA repair by targeting the MRN complex, which would favor error prone non-homologous end joining DNA repair pathway and perhaps increase the risk of cell transformation [174].

Early studies which examined molecular clones that failed to express ORF-II mRNA or produced truncated forms of p30 indicated that ORF II was not required for virus replication in cell culture or *in vitro* T-cell transformation [175]. In contrast, our lab has demonstrated that HTLV-1 molecular clones that lack p30 expression do not establish infection in a rabbit model of HTLV-1 infection [124]. In conclusion, p30 is a multifunctional protein important in multiple transcriptional and post-transcriptional phases of HTLV-1 replication and appears to be required for the virus to establish a productive infection in the host.

### 6.4. HBZ

HTLV-1 expresses two mRNA products from the 3′ LTR of the complementary strand of the virus genome of 2.6 kb and 2.9 kb in length [176]. The protein produced from these anti-sense transcripts has been named HTLV-1 bZIP factor or HBZ. HBZ is composed of 209 amino acids and contains a NLS, amino terminal transcriptional activation domain and a carboxyl terminal leucine zipper motif [177,178].

Recent *in vitro* and *in vivo* studies have elucidated the function of HBZ in the virus life cycle. *In vitro* studies demonstrated that the carboxyl terminal truncations of HBZ did not affect virus replication and immortalization. Deletion of HBZ expression, however, from infectious molecular clones of HTLV-1 resulted in decreased in proviral load and antibody responses in the rabbit model [154] and knock down of HBZ expression reduces tumor growth in a mouse model of ATL [179]. The inability of HTLV-1 to persistently infect rabbits without HBZ expression is most likely attributed to the lost of interactions between HBZ and important viral and cellular proteins. HBZ has been shown to interfere with Tax mediated viral transactivation of the LTR in a TRE dependant manner [177]. HBZ can form heterodimers with CREB-2, which prevents recruitment to the TRE and CRE transcriptional regions [177]. This implies that HBZ might be a negative regulator of viral transcription.

In addition to CREB-2, HBZ can form dimers with other proteins, which are important for transcription. HBZ interacts with JunD, JunB and c-Jun [153,180]. Dimerization between HBZ and JunD or JunB increases transcriptional activity of both transcription factors [153,180]. However, dimerization between c-Jun and HBZ results in a decrease in c-Jun transcriptional activity [153]. Studies have also shown HBZ can impair the DNA binding of AP-1, another important transcription factor [181]. Mice transgenic for HBZ have increased proliferation of CD4+Foxp3+ T(reg) cells but have reduced ability to suppress other lymphocyte proliferation perhaps through interaction with Foxp3 and NFAT [182]. Collectively, these studies have elucidated potential findings of HBZ in the context of the HTLV-1 life cycle; however more studies are required to completely understand the role of HBZ in the natural infection and in the pathogenesis of HTLV-1-mediated diseases.

## 7. Animal Models to Evaluate Viral Determinants of HTLV-1 Transmission and Spread

Since the discovery of HTLV-1, a number of animal models of HTLV-1 transmission and spread have provided fundamental information about viral and host determinants of infection [183]. Rabbits [184,185], some nonhuman primates [186,187] and rats [188,189] can all be infected with HTLV-1 and have been utilized to monitor the virus spread, determine immune responses against the infection and in the development of vaccines against the viral infection. These animal models of HTLV-1 infection or disease have been recently reviewed [183]. In evaluating an appropriate animal model of HTLV-1 infection or disease, it is important to differentiate animal models that test individual genes of the virus from those that place the gene under the typical control of HTLV-1 in context of a complete genome. To accurately model HTLV-1 determinants of viral transmission and spread, the model system should: (1) be tested in context of the complete viral genome, (2) test mutant viral clones concurrent with positive controls that demonstrate infectivity with parameters (e.g., proviral loads) similar to those used to monitor humans infected with the virus, (3) reproducibly elicit persistent infections with widespread distribution of the virus as seen in humans infected with HTLV-1, and (4) ideally be economical and easily monitored e.g., animals with blood volumes that allow multiple serial measurements.

**Figure 3:**
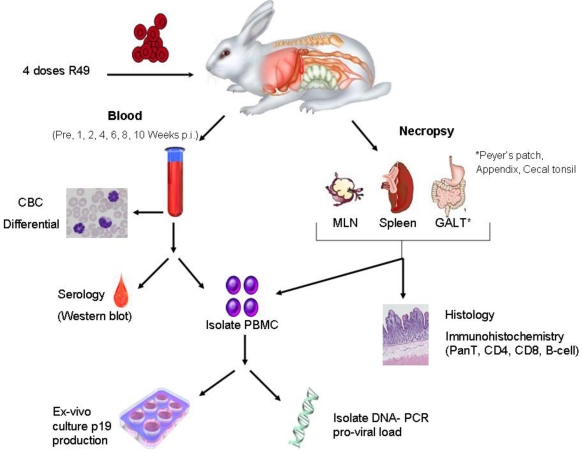
Rabbit model of HTLV-1 transmission demonstrates a reproducible system to produce persistent infections with widespread distribution of the virus similar to humans in an economical and easily monitored model. Example showing inoculation of HTLV-1-transformed cell line (R49) with ACH proviral clone [204]. Determinants of viral transmission and spread can be measured in context of the complete viral genome with the ability to test mutant viral clones concurrent with positive controls that demonstrate infectivity with parameters (e.g., proviral loads).

Rabbits have been used extensively as a model of HTLV-1 infection in humans because of the ease and consistency of transmission of the viral infection in this species (Figure 3). Infectivity of rabbits was first demonstrated in the mid-1980s using intravenous inoculations of the MT-2 cell line [184], a T-cell leukemia cell line established from a patient with ATL, and with the Ra-1 cell line [190], a rabbit lymphocyte cell line derived from co-cultivation of rabbit lymphocytes with MT-2 cells. These early studies verified routes of transmission (e.g., blood, semen, milk) for the virus infection [191–196]. Importantly, studies using the rabbit model of HTLV-1 have provided accurate data to estimate the number of cells capable of transmitting the virus infection [193] and effective means to prevent the transmission of the virus [62,193,197–199]. Rabbit-based studies defined the sequential development of antibodies against the virus infection [200]. Immunization of rabbits with synthetic peptides verified immunodominant epitopes of the viral envelope protein (Env) [201,202] and defined regions of Env important for antibody dependent cell-mediated cytotoxicity [203].

Infectious molecular clones of HTLV-1 were first developed in the mid-1990s [48,205,206] and opened the door for testing viral determinants in context to the viral infection *in vivo* [207]. Subsequently, ACH clones with mutations within the open reading frames encoding the HTLV-1 accessory proteins, p12, p13, and p30, were generated [175], and used in rabbits to demonstrate the necessity of these accessory proteins for establishment of infection and maintenance of proviral loads [121,122,124,162]. The roles of Rex and HBZ in viral transmission and spread have been tested using the ACH clone in rabbits [116,154]. Using ACH wild type virus immortalized rabbit T-cells in an intravenous infection model, HTLV-1 was found to accumulate in primary lymphoid and gut-associated lymphoid compartments in rabbits and was associated with an early lymphocytosis [204]. These data are consistent with a variety of human studies that support emerging evidence that HTLV-1 promotes lymphocyte proliferation preceding early viral spread in lymphoid compartments to establish and maintain persistent infection. Immunosuppressive treatment prior to HTLV-1 infection in the rabbit model (modeling case reports in human transplant patients exposed to contaminated blood products) reveal enhanced early viral expression compared to untreated HTLV-1-infected rabbits, and altered long-term viral expression parameters. However, this same treatment one week post infection diminished HTLV-1 expression for greater than 10 weeks, interfering with typical viral loads [208]. This type of study extends studies of chronically infected humans that indicate that immunologic control is a key determinant of viral persistence [209–211]. The rabbit model clearly demonstrates the sequential control during early HTLV-1 spread and offers a tractable model to test therapeutic strategies during mucosal transmission.

Mouse models of ATL, which typically are focused on xenografts to test anti-cancer compounds or transgenic mice to test the properties of individual oncoproteins, have been recently reviewed [212]. As a complete genome inoculated in a cell-associate form, HTLV-1 consistently infects rabbits [184,185], some non-human primates [186,187], and to a lesser extent rats [188,189]. Viral transmission in mice has produced inconsistent infections with limited virus expression in tissues and thus do not provide a reliable system to test virus spread in the host [213–216]. Non-human primates have been infected with HTLV-1 and a number of species have natural infections with various strains of simian T-lymphotropic virus infection type 1 [217–219]. The squirrel monkey has been successfully infected with HTLV-1 and has been used for HTLV-1 vaccines [24,220–222]. In a limited study, HTLV-1 mutant molecular clones were tested for their ability to replicate in dendritic cells and *in vivo* in rabbits and macaques [126]. Some strains of rats have been utilized as a model of HTLV-1-associated myelopathy/tropical spastic paraparesis (HAM/ATL), the neurologic disease associated with the viral infection [189,223–227]. While rats have been used to test the role of cell-mediated immunity to the infection [224,228], the reproducibility of the infection has been questioned [188]. Sheep infected with bovine leukemia virus are a reliable model of lymphoma and have provided knowledge of viral genetic determinants of viral spread *in vivo* [229–231].

Experimental infection of F344 rats with HTLV-1 was first established in 1991 [189]. Subsequently, differences in the response of various rat strains to HTLV-1 infection were demonstrated [188,227,232]. Wistar-King-Aptekman-Hokudai (WKAH) rats develop spastic paraparesis with degenerative thoracic spinal cord and peripheral nerve lesions several months following inoculation [227,232]. The neurologic lesions of WKAH-infected rats were predominantly characterized by macrophage infiltrates, which differs from that seen in humans [233].

Experimental HTLV-1 infection has produced persistent infections in several species of non-human primates inoculated with MT-2 cells, Ra-1 cells, or autologous HTLV-1-infected cell lines [234–236]. HTLV-1 immortalized cell lines from Squirrel monkeys have been used in this species to document peripheral lymphocytes, spleen and lymph nodes as major reservoirs for the virus during the early phase of infection [221,237,238]. HTLV-1 infection in squirrel monkeys appears to be dependent on early reverse transcription of the virus genome, followed by clonal expansion of infected cells [24]. HTLV-1 inoculated rhesus macaques appear to develop a higher incidence of arthritis, uveitis, and polymyositis [239]. In addition, pig-tailed macaques that died naturally at 35 to 82 weeks post-inoculation with ACH HTLV-1 molecular clone HTLV-1 exhibited lymphopenia, arthropathy, and diarrhea [240]. While a promising model species, the cost of maintenance of non-human primates have limited more extensive use of this model to test HTLV-1 determinants of virus transmission.

## 8. Conclusions

Human T-lymphotropic virus type-1 (HTLV-1) has evolved to spread from mother to child through breast milk or via blood transfusion and is associated with adult T-cell leukemia/lymphoma (ATL), and a variety of lymphocyte-mediated disorders such as HTLV-1-associated myelopathy/tropical spastic paraparesis (HAM/TSP). The complex retrovirus contains typical gag, pol, and env genes, but also unique nonstructural proteins encoded from the pX region that are essential for viral spread *in vivo*. Infectious molecular clones of the virus have allowed testing of viral genes in the transmission and spread of the virus. Further studies, however, of specific functional motifs of key viral gene products in context to mucosal transmission are needed to determine the interplay of host defenses and these viral determinants in viral transmission and spread of HTLV-1.

## Figures and Tables

**Table 1:** Human T-lymphotrophic virus type-1 (HTLV-1) -associated diseases and syndromes.

**Disease or Syndrome**	**Clinical Characteristics and Pathologic Outcomes**
Adult T-cell leukemia/lymphoma (ATL)	❖ Four classifications based on clinical signs include: asymptomatic, pre-leukemic, chronic smoldering, and acute ❖ Clinical symptoms may include malaise, fever, lymphadenopathy, hepatosplenomegaly, hypercalcemia, lytic bone lesions, elevated lactate dehydrogenase, increased interleukin 2 receptor in serum, lymphomatous skin infiltrates, jaundice, weight loss, and various opportunistic infections, such as *Pneumocystis carinii* ❖ Aggressive malignancy of T-lymphocytes, characterized by multiple distinct cell surface markers, including CD3+/CD4+/CD8−/CD25+/HLA−DR+ T-cells ❖ Leukocytosis may include atypical cell morphology, multilobulated nucleus referred to as “flower cells” ❖ Diagnostic criteria include HTLV-1 seropositivity, leukocytosis, increased serum levels of IL-2 receptor and LDH, demonstration of neoplastic T-cells with polylobulated nuclear morphology (“flower cells”), and clonally integrated HTLV-1 genomes within the chromosomes of neoplastic lymphocytes
HTLV-1-Associated Myelopathy/Tropical Spastic Paraparesis (HAM/TSP)	❖ Spasticity lower extremities, hypereflexia, muscle weakness, and sphincter disorders, including dysfunction of the urinary bladder and intestines; clinically may overlap with multiple sclerosis ❖ Progressive chronic myelopathy, with preferential damage of the thoracic spinal cord ❖ Early lesion development characterized by infiltrates composed predominantly of CD4+ T-cells, and macrophages with detectable levels of HTLV-1 *tax* RNA in lesions ❖ Characterized by multiple white matter lesions in both the spinal cord and the brain involving perivascular demyelination and axonal degeneration; rarely, cerebellar syndrome with ataxia and intention tremor ❖ Late lesions (>4 years) predominantly CD8+ T-cells with less *tax* RNA ❖ Cerebrospinal fluid contain high levels of proinflammatory cytokines, including IFN-γ, TNF-α, IL-1, and IL-6, as well as increased numbers of activated lymphocytes
HTLV-1-associated Dermatitis	❖ Chronic eczema with refractory *Staphylococcus aureu*s or beta-hemolytic streptococcus infections ❖ Described in Jamaican children as “infectious dermatitis” ❖ Patients frequently develop HAM/TSP later in life and may have episodes of severe anemia
Ocular Lesions	❖ HTLV-1 infection associated in endemic regions with uveitis, keratoconjunctivitis sicca, and interstitial keratitis ❖ Chronic course in children may result in retinal degeneration
Inflammatory Arthropathy and Polymyositis	❖ Chronic polymyositis associated with HTLV-1 may be presented with neuropathy, joint swelling, chest pain, and dyspnea ❖ Japanese patients in regions endemic for HTLV-1 infection may present with chronic inflammatory arthropathy or polymyositis ❖ Similar lesions have been reproduced in transgenic mouse and rat models
